# Association of Sociodemographic, Socioeconomic and Lifestyle Characteristics with Low Protein and Energy Intake in the Healthy Swiss Population

**DOI:** 10.3390/nu15092200

**Published:** 2023-05-05

**Authors:** Roxana Wimmer, Andrea Audétat, Julia Binggeli, Philipp Schuetz, Nina Kaegi-Braun

**Affiliations:** 1 Medical University Department, Division of Endocrinology, Diabetes and Metabolism, Kantonsspital Aarau, 5001 Aarau, Switzerland; philipp.schuetz@ksa.ch (P.S.); nina.kaegi@ksa.ch (N.K.-B.); 2 Medical Faculty, University of Basel, 4056 Basel, Switzerland; andrea.audetat@ksa.ch (A.A.); julia.binggeli@ksa.ch (J.B.)

**Keywords:** protein intake, dietary intake, energy intake, sociodemographic, socioeconomic, lifestyle

## Abstract

A balanced diet has the goal of providing adequate amounts of different nutrients to promote and maintain physical and psychological health. Our aim was to study the association between different sociodemographic, socioeconomic and lifestyle factors and low energy or protein intake among the Swiss population. This is a cross-sectional cohort study based on the national nutritional survey “MenuCH”, which is the first representative, detailed assessment of dietary habits in the adult Swiss population conducted in 2014/2015. We compared the mean protein and caloric intake based on two 24 h recall nutritional assessments with current recommendations based on resting metabolic rate calculation and DACH guidelines. A total of 1919 participants with a median age of 46 years and 53% females were included. Overall, 10.9% and 20.2% of participants had an energy and protein intake, respectively, below the dietary reference values. However, a high income (>9000 CHF per month) reduced the risk of low energy intake (OR 0.49 [0.26–0.94], *p* = 0.032), obesity (OR 6.55 [3.77–11.38], *p* < 0.01), and living in a household with children (OR 2.1 [1.15–3.85], *p* = 0.016) was associated with higher risk. Regarding low protein intake, the most important risk factors were an age group of 65–75 years (OR 2.94 [1.57–5.52], *p* = 0.001) and female gender (OR 1.73 [1.15–2.6], *p* = 0.008). Regular meat consumption reduced the risk of low protein intake (OR of 0.23 (0.1–0.53), *p* = 0.001). Within this survey, several socio-economic and lifestyle factors were associated with low energy and protein intake in the healthy Swiss population. A bunderstanding of these factors may help to reduce the risk of malnutrition.

## 1. Introduction

A balanced diet should provide adequate amounts of nutrients to promote and maintain physical and psychological health and to prevent malnutrition. In consequence, many nutrition scientific societies provide dietary reference values (DRV) for healthy individuals [1,2,3,4]. They are used for many goals, including the formulation of specific nutritional recommendations and food-based dietary guidelines to different identified populations, to serve as the basis for nutritional information on food labels and to define nutrition policies to help consumers make positive choices for a balanced diet [5].

While the pathophysiology of malnutrition is highly complex and involves several pathways, there is convincing evidence that low energy and protein intake causes malnutrition, particularly in the population of elderly frail patients [6]. Therefore, understanding the factors that affect nutritional intake is highly important. Particularly, several studies conducted in Europe have shown the influence of sociodemographic, socioeconomic and lifestyle factors such as overweight/obesity, education, and older age on the individual protein and caloric intake [7,8,9,10,11,12].

According to the “European Society of Parenteral and Enteral Nutrition” (ESPEN) classification [7], there are two main entities of malnutrition: “disease-related malnutrition” (DRM) and “malnutrition without disease”, the latter including “socioeconomic” and “hunger-related” malnutrition. For both entities, total energy intake and protein intake are important factors. DRM is prevalent (about 30% of hospitalized patients) and has been studied extensively in recent years. However, the literature about “malnutrition without disease”, especially “socioeconomic malnutrition” in high-income countries, is scare.

Until now, little has been known about the prevalence and the predictors of low protein and caloric intake in the healthy Swiss population. Herein, our aim was to use a population-based cohort of healthy Swiss individuals to investigate the prevalence of low energy and protein intake, defined by basal metabolic rate as well as DRV, and identify the sociodemographic, socioeconomic and lifestyle factors are associated with low energy and protein intake.

## 2. Materials and Methods

### 2.1. Study Design and Study Population

For this study, we analyzed data from menuCH, a population-based cross-sectional survey study conducted between January 2014 and February 2015. This survey was the first and last representative national sample that collected detailed information regarding the protein and energy intake of the healthy Swiss population. Data were obtained from a stratified random sample of residents age 18–75 years and representative of seven administrative regions (Lake Geneva region, Midlands, Northwest Switzerland, Zürich, Eastern Switzerland, Central Switzerland and Ticino) distributed over three main language regions (German, French, Italian).

Recruitment was carried out by mail or phone, and those who agreed to participate were invited to the study center for a personal interview and nutritional assessment. Diet was assessed by 24 h dietary recalls (24HDR). Additional data, such as sociodemographic, socioeconomic and lifestyle factors, as well as anthropometric measurements, were collected. A second 24HDR was performed by phone. Detailed information about the menuCH survey was published previously [13,14]. As in the original trial, we included all patients with complete data of both 24 HDR but we excluded participants who were pregnant, on lactation or currently on a weight loss diet because of their different intake goals.

### 2.2. Calculation Protein and Energy Intake Classification

We used mean energy and protein intake of the two 24HDR for our analysis. We calculated resting metabolic rate (RMR) by means of the Mifflin St. Jeor formula [15]. No activity level multiplication factor was used. According to their RMR, participants were classified as “energy intake above RMR” or “energy intake below RMR”.

Adequate protein intake was defined according to the Swiss, German and Austrian guidelines (D-A-CH) [16]: >0.8 kg per kg bodyweight for individuals <65 years and >1.0 per kg bodyweight if >65 years. For patients with BMI > 25 kg/m^2^, we limited the protein goal to corrected body weight for BMI 25 kg/m^2^.

### 2.3. Sociodemographic Factors

The participants received a questionnaire containing information on their sociodemographic and socioeconomic factors, and data were checked for completeness and accuracy by the dieticians at the study center during the personal interview.

The following sociodemographic factors were observed: age, sex, nationality, language region, household type, and marital status. Age was calculated from the self-reported birth date and was categorized into 4 groups (18–29, 30–44, 45–59, 60–75 years). A distinction was made between participants with nationality “Swiss” and “Non-Swiss”. Participants with double citizenship were classified as “Swiss” if one of their nationalities was “Swiss”. The language region was determined according to the participants’ canton of residence (German-/French-/Italian-speaking). Household type was assessed in detail regarding the people living in the same household. We subsumed those groups into two main household types: residents living with or without children. For marital status, we stratified the study population into subjects who were married or in a registered relationship vs. individuals with the status of single.

### 2.4. Socioeconomic Factors

Education and household income were the two socioeconomic variables of interest in our study. The education status (highest completed degree) was grouped into primary (no compulsory schooling, compulsory schooling not finished, compulsory schooling finished), secondary (internship and any other education, federal professional diploma, full-time professional school, apprenticeship with diploma, professional school, vocational high school, former school to become a teacher, high-level apprenticeship, high school) and tertiary degrees (high-level federal professional diploma, high technical school, high-level professional school, bachelor in pedagogy to become a teacher, university of applies sciences, university degree, Ph.D.). Participants were asked to classify their net household income into six different categories, which we summarized into three income groups (<6000 CHF/month, 6000–9000 CHF/month, >9000 CHF/month).

### 2.5. Lifestyle Factors

We focused on the following lifestyle factors: smoking status, alcohol consumption, self-reported physical activity, meat consumption and whether the food was consumed mostly at home or regularly outside. Smoking status was dichotomized into smokers and non-smokers. Former smokers were classified as (current) non-smokers. Alcohol consumption was categorized into two groups according to their alcohol intake during the days the survey took place. No or low alcohol consumption was defined as consuming less than 10 g of alcohol per day for women and less than 20 g per day for men, as recommended by the WHO in 2001 [17]. Alcohol consumption above these recommendations was classified as high alcohol consumption. Participants had to report their physical activity according to the short version of the International Physical Activity Questionnaire (IPAQ) and were divided into two groups according to the WHO recommendations for physical activity [18,19].

Participants who stated vegetarian or vegan eating habits or avoided meat and fish were categorized as “no meat consumption”. The amount of meat consumed was not considered.

We defined eating outside home regularly as consuming more than four meals per week that were not prepared at home (e.g., meals from a restaurant or take-away). Not included were meals eaten at a home other than the participant’s, as those meals are likely to be homecooked.

### 2.6. Weighting Strategy

Applying the results of the survey to the entire Swiss population, we used weighting factors to correct for the sampling design and non-response. Results were weighted for age, sex, marital status, language region, nationality and household size. Additionally, data were corrected for the uneven distribution of the interviews over seasons and weekdays [20].

### 2.7. Statistical Analysis

We used descriptive statistics, reported means and standard deviations to determine continuous, number and percentages, respectively, for binary or categorical variables. To assess the association of different factors with low energy and protein intake, we used uni- and multivariable logistic regression models. Missing variables were treated as missing.

Our analyses were conducted using either Stata version 15 or Stata version 17 (StataCorp, College Station, TX, USA). *p* < 0.05 was considered statistically significant.

## 3. Results

### 3.1. Recruitment

**Figure 1:**
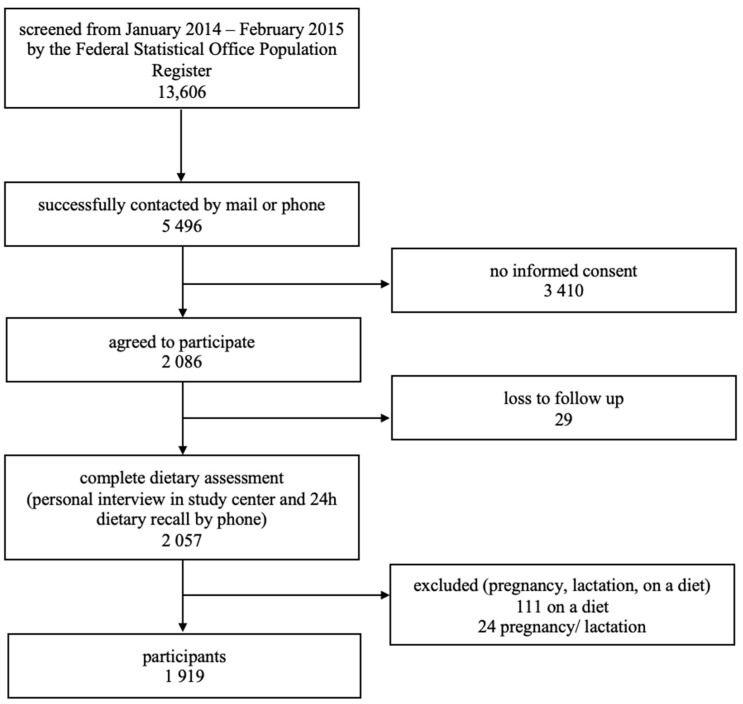
Trial profile.

In the menuCH survey, 13,606 subjects, age 18–75 years, were randomly selected using data from the population register of the Federal Statistical Office. A total of 5496 were successfully contacted by mail or phone and 2086 agreed to participate in the study (38% participate rate). Among the 2086 participants, 2057 had a complete dietary assessment and were finally included in the “MenuCH” study. From the 2057 participants, we excluded 138 persons who were either pregnant, lactating or on a weight-loss diet. A total of 1919 participants were finally included in our analysis (Figure 1).

### 3.2. Population Characteristics

Table 1 summarizes the overall characteristics of the unweighted sample. The mean age was 46 years (ranging from 18 to 75 years) and 53% were female. The majority were Swiss citizens (87%) and residents of the German-speaking region of Switzerland. A total of 63% did not have children and the majority lived with at least one other person (85%). More than half were married (55%). Most of the participants had a secondary or tertiary education and income classes were almost equally distributed. A total of 55.4% had a BMI between 18.5 and 24 kg/m^2^ and most of the participants were non-smokers (78%). A total of 2% of participants reported not eating meat.

### 3.3. Predictors of Energy Intake below Resting Metabolic Rate (RMR)

In a first step, we investigated factors associated with low energy intake (Table 2). Overall, 10.9% of the participants had an energy intake below their RMR. We found several factors associated with low energy intake, including high income (>9000 CHF per month) and high alcohol consumption, which were both associated with a lower risk for low energy intake (OR 0.49 [0.26–0.94], *p* = 0.032 and OR 0.61 [0.38–0.97], *p* = 0.037). Higher risk for low energy intake was found for overweight and obesity. After stratification by sex, the association of BMI and low energy intake was similar in both groups, while high income was only significantly associated with sufficient energy intake in male participants (OR 0.27 [0.12–0.62], *p* = 0.002). Additionally, male current smokers had a higher risk of not reaching their RMR. In female participants, Swiss nationality was associated with adequate energy intake, whereas living in a household with children increased the risk of low energy intake (OR 2.1 [1.15–3.85], *p* = 0.016) (Supplementary Table S1).

### 3.4. Predictors for Not Meeting Protein Dietary Reference Values (DRV)

In a second step, we investigated factors associated with low protein intake (Table 3). A total of 322 (20.2%) participants had a protein intake according to our definition. In the regression analysis, the age group of 65–75 years showed an almost three-fold risk of low protein intake (OR 2.94 [1.57–5.52], *p* = 0.001). Female individuals were at higher risk of not meeting their protein DRV (OR 1.73 [1.15–2.6], *p* = 0.008). Overall, there was no significant association with socioeconomic factors such as education and income. Overweight and obese participants met their DRV less often, while the opposite was found for individuals which were drinking alcohol (OR 0.63 [0.42–0.93], *p* = 0.019). However, the strongest protective association was found for meat consumption, with an OR of 0.23 (0.1–0.53), *p* = 0.001.

**Figure 2:**
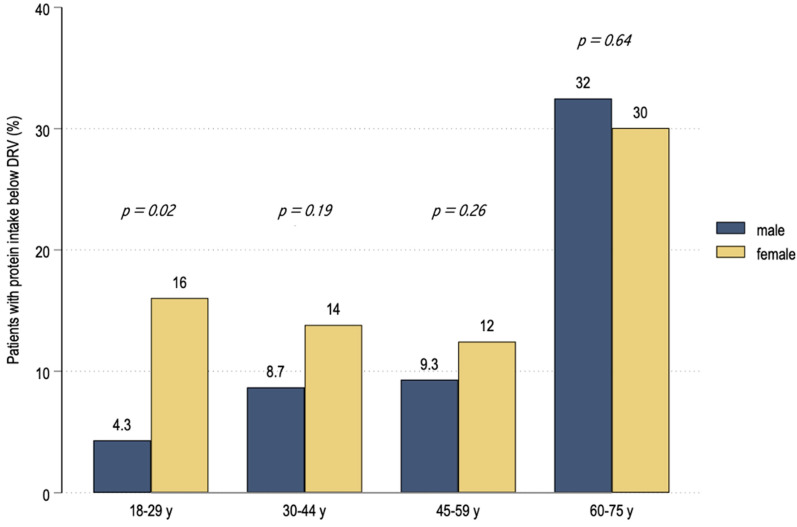
Percentages of participants with protein below DRV stratified by age and sex.

**Figure 3:**
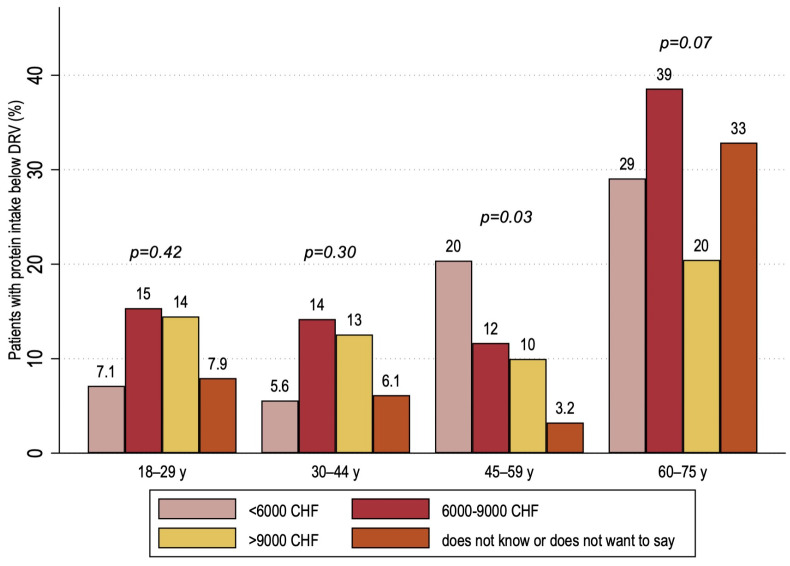
Percentages of participants with protein below DRV stratified by age and income.

After stratification by age groups, we found that the lower protein intake of women was mainly present in young participants, while there was no significant difference between the age groups from 45 to 75 years (Figure 2, Supplementary Table S2). In addition, in the age group of 45–59 years, we found low protein intake significantly more often in the lowest income class (Figure 3). However, results were not statistically significant in the regression model of the subgroup analysis.

## 4. Discussion

Overall, 10.9% of the participants had a lower energy intake compared to their resting metabolic rate, and 20.2% had a lower protein intake compared to the recommended dietary reference value.

The risk of a protein intake below DRV was increased almost three-fold in the age group from 65 to 75 years. Previous studies already showed that, with higher age, the risk of low protein intake increases in healthy as well as hospitalized patients [21,22,23,24,25]. Potent risk factors may be reduced appetite, difficulties in chewing and intolerance of certain foods. These factors contribute, in combination with a lack of physical activity, to an age-related decline in skeletal muscle [26].

Young female individuals were more likely to have insufficient protein intake. This should be seen in the context of meat consumption, which has been shown to be higher in the younger age groups and lower in female individuals [27,28].

In our analysis, meat consumption was a strong protective factor against low protein intake. However, during the survey period, the number of no-meat eaters was very low (2.0%) and the main source of dietary protein was animal (Supplementary Figure S1), which contains more protein per calorie than plant-based proteins [27,29,30]. However, alternative protein sources are more available at present, as vegetarianism/veganism gained popularity in the last year.

A protective factor against low energy and protein intake was high alcohol consumption. This result should be interpreted carefully, because the alcohol consumption was based only on the two 24 h dietary recalls and high alcohol consumption might correlate with an extensive meal and meat intake. Additionally, alcohol has a high caloric value of about 7 kcal/g [31].

Interestingly, a higher BMI was associated with low energy intake, as well as protein intake, even though we excluded participants who stated they were on a weight loss diet and set protein goals adjusted to a body weight with a BMI 25 kg/m^2^. This might be due to an underreporting of caloric intake or alternative macronutrient intake, such as fat. As for energy intake, an imprecise calculation of RMR could be another possible explanation. In comparison to other formulas, the Mifflin–St Jeor formula performed best in obese individuals; however, the measurement of RMR is still preferred [32].

Marital status was found to influence dietary intake [33] and to be associated with a lower intake of macronutrients (energy, protein) [34]. Furthermore, Pearson et al. [35] showed that individuals living in a single household had a lower protein intake in comparison to those who lived with others. Our results showed no significant association and are in line with another study by Kyung et al. [36].

Eating habits differ between smokers and non-smokers concerning dietary behaviors [37,38] and source of protein intake [39]. Nicotine has an anorectic effect on appetite, resulting in lower total food consumption and, therefore, the possible inclusion of protein-rich foods [40]. However, in our study, there was a trend towards less caloric intake in smokers, but no association with protein intake.

Switzerland represents a unique setting, consisting of three main language regions (German-speaking, French-speaking, Italian-speaking). Dietary habits differ according to cultural heritage, geographic location, and agricultural tradition. Pestoni et al. [41] used the Alternate Healthy Eating Index (AHEI) to compare diet quality across Switzerland`s main language regions. Participants from the French- and Italian-speaking regions outperformed those from the German-speaking region. Regarding protein intake by dairy products or meat, the German-speaking region is characterized by a higher intake compared to the French- and Italian-speaking regions [27,42]. Overall, despite these regional differences, the incidence of low energy or protein intake is well-balanced, as our results showed no significant differnce between the language regions.

Data on the relationship between socioeconomic status and protein intake are inconclusive. Friel et al. [43] was able to demonstrate that dietary protein intake varies between socioeconomic groups. Interestingly, according to Si Hassen et al. [11], individuals with a low education level and higher income showed a higher protein intake. While the absolute intake of proteins seems to be higher, in another analysis, lower educated individuals consumed less protein as a percentage of their total energy [12]. In our cohort, participants with a high income had a lower risk of low energy intake, while no differences have been revealed concerning protein intake in terms of educational attainment or household income.

In this analysis, we focused on low energy and protein intake in the context of malnutrition. Nevertheless, overnutrition is an even more important topic in industrialized countries. Therefore, we also assessed the association between high protein intake (defined as >2 g/kg/d) and sociodemographic, socioeconomic and lifestyle factors (Supplementary Table S3). A total of 5.63% of the participants had a high protein intake with risk factors such as young age, male gender, low education and BMI < 18.5 kg/m^2^. We did not assess high energy intake because we lacked the variables to define high intake individually.

A strength of our study is that the data were obtained from the largest nation-wide nutritional survey at present. Statistical weighting of the data was performed, correcting for changes in the study population due to non-response, and for the uneven distribution of dietary reporting across the seasons and weekdays. Based on a national, stratified, random, population-based sample, the study design allows for a generalization of the results at a national level. Dietary intake was assessed using 24 h dietary recalls by trained dieticians using the automated and validated GloboDiet^®^ Software (formerly EPIC-Soft^®^, version CH-2016.4.10, International Agency for Research on Cancer (IARC), Lyon, France), which meets European standards in food consumption surveys. The use of high-quality assessment tools allows for a limited probability of reporting bias [44].

We acknowledge that the study has several limitations. First, no causal inferences can be drawn due to the cross-sectional design of the study. Persons above the age of 75 years and those who do not speak either German, French or Italian were excluded. Additionally, some information was missing, for example, income data were not complete for the whole study population, which could lead to selection bias. The dietary assessment is based on self-reported information, which could have led to reporting and underreporting bias due to social desirability. It is likely that participants underreported energy intake and food known to be unhealthy while overreporting healthier food, especially in a 24 h dietary recall [45].

Second, the survey took place from the years 2014 to 2015. In recent years, diet and food consumption in Switzerland have changed substantially as vegetarianism and veganism have gained popularity. The consumption of plant-based substitutes is widespread in the population, regardless of whether a person identifies as vegetarian or vegan [46]. Further studies are needed to assess the current situation.

Third, with our analysis, we assessed energy intake by using cut-off values for adequate energy and protein intake. Regarding energy, we used RMR and did not consider the activity factor; therefore, our calorie threshold is rather low. In the case of protein, we used DRV, a scientific reference for professionals, who use it when setting nutrient goals for populations or recommendations for individuals [4]. We did not consider other individual factors for the calculation of the macronutrient goals, and although we used rather low thresholds for both, we cannot draw conclusions about the impact of a low intake on health outcomes. However, there are studies showing the consequences of low protein intake in elderly men, reporting a modest increase in all-cause mortality [47,48].

Identifying factors which are associated with a low protein and low caloric intake could help define risk populations, and improve nutritional recommendations as well as the malnutrition screening process, which is usually based on nutritional history (e.g., weight loss, nutritional intake), anthropometric measurements (e.g., BMI) and disease-specific factors (e.g., disease severity).

## 5. Conclusions

In conclusion, within this survey, several socioeconomic and lifestyle factors were associated with low energy and protein intake in the healthy Swiss population. The causes of these deficits might differ between groups. Anatomical changes, as in the case of older age, different eating habits depending on gender or lifestyle factors and the financial situation, might play a role.

A better understanding of these factors may help to promote a balanced diet and reduce the risk of malnutrition in the elderly population.

To identify populations where early nutritional prevention is useful, there is a compelling need for long-term studies that look at today’s healthy population with a low macronutrient intake and examine whether these individuals suffer from health problems or are at higher risk of developing malnutrition in the case of acute or chronic disease.

## Figures and Tables

**Table 1:** Baseline characteristics of overall, unweighted population.

	Total
	N = 1919
Mean Intake	
Protein	1.22 g/kgBW
Energy	2183 kcal
Sociodemographic factors	
Age	
18–29 years	374 (19.5%)
30–44 years	470 (24.5%)
45–59 years	596 (31.1%)
60–75 years	479 (25.0%)
Sex	
Male	900 (46.9%)
Female	1019 (53.1%)
Nationality	
Non-Swiss	244 (12.7%)
Swiss	1675 (87.3%)
Language Region	
German speaking	1249 (65.1%)
French Speaking	473 (24.6%)
Italian Speaking	197 (10.3%)
Household Type	
Without Children	1214 (63.3%)
With Children	702 (36.6%)
Missing	3 (0.2%)
Marital Status	
Not married	868 (45.2%)
Married	1048 (54.6%)
Missing	3 (0.2%)
Socioeconomic factors	
Education, Highest Degree	
Primary	81 (4.2%)
Secondary	904 (47.1%)
Tertiary	931 (48.5%)
Missing	3 (0.2%)
Gross Household Income	
<6000 CHF/month	328 (17.1%)
6000–9000 CHF/month	391 (20.4%)
>9000 CHF/month	662 (34.5%)
Does not know/refuses to say	224 (11.7%)
Missing	314 (16.4%)
Lifestyle factors	
BMI	
<18.5 kg/m^2^	50 (2.6%)
18.5–24 kg/m^2^	1063 (55.4%)
25–29 kg/m^2^	579 (30.2%)
30–34 kg/m^2^	172 (9.0%)
35–39 kg/m^2^	40 (2.1%)
>45 kg/m^2^	9 (0.5%)
Missing	6 (0.3%)
Self-Reported Physical Activity	
Low	736 (38.4%)
Moderate	585 (30.5%)
High	551 (28.7%)
Missing	47 (2.4%)
Smoking Status	
Never	856 (44.6%)
Former	639 (33.3%)
Current	420 (21.9%)
Missing	4 (0.2%)
Alcohol Consumption	
No or low alcohol consumption	525 (27.4%)
Higher consumption	1394 (72.6%)
Meat Consumption	
Yes	1881 (98.0%)
No	38 (2.0%)
Eating Habits	
≤4 meals outside home	1010 (52.6%)
>4 meals outside home	909 (47.4%)

Abbreviation: CHF: Swiss franc, BW: body weight.

**Table 2:** Predictors for energy intake below resting metabolic rate (RMR).

	Energy Intake above RMR	Energy Intake below RMR		
	N = 1709	N = 210		
Sociodemographic factors				
			OR univariate (95% CI), *p*-value	OR multivariate (95% CI, *p*-value
Age				
18–29 years	335 (19.6%)	39 (18.6%)	reference	
30–44 years	411 (24.0%)	59 (28.1%)	0.87 (0.5 to 1.51), *p* = 0.611	0.76 (0.37–1.56), *p* = 0.453
45–59 years	530 (31.0%)	66 (31.4%)	0.93 (0.54 to 1.61), *p* = 0.799	0.78 (0.39–1.55), *p* = 0.483
60–75 years	433 (25.3%)	46 (21.9%)	0.92 (0.49 to 1.7), *p* = 0.784	0.65 (0.27–1.58), *p* = 0.339
Sex				
Male	794 (46.5%)	106 (50.5%)	reference	
Female	915 (53.5%)	104 (49.5%)	0.73 (0.5 to 1.05), *p* = 0.087	1.26 (0.81–1.97), *p* = 0.306
Nationality				
Non-Swiss	211 (12.3%)	33 (15.7%)	reference	
Swiss	1498 (87.7%)	177 (84.3%)	0.68 (0.42 to 1.09), *p* = 0.109	0.66 (0.38–1.14), *p* = 0.137
Language Region				
German speaking	1134 (66.4%)	115 (54.8%)	reference	
French Speaking	416 (24.3%)	57 (27.1%)	1.17 (0.78 to 1.76), *p* = 0.444	1.16 (0.72–1.87), *p* = 0.551
Italian Speaking	159 (9.3%)	38 (18.1%)	1.89 (1.18 to 3.03), *p* = 0.008	1.42 (0.79–2.56), *p* = 0.242
Household Type				
Without Children	1095 (64.1%)	119 (56.7%)	reference	
With Children	612 (35.8%)	90 (42.9%)	1.2 (0.82 to 1.75), *p* = 0.353	1.41 (0.89–2.24), *p* = 0.147
Marital Status				
Not married	775 (45.3%)	93 (44.3%)	reference	
Married	932 (54.5%)	116 (55.2%)	0.86 (0.59 to 1.25), *p* = 0.433	1.04 (0.61–1.75), *p* = 0.896
Socioeconomic factors				
Education, Highest Degree				
Primary	66 (3.9%)	15 (7.1%)	reference	
Secondary	795 (46.5%)	109 (51.9%)	0.52 (0.23 to 1.16), *p* = 0.109	0.79 (0.35–1.79), *p* = 0.575
Tertiary	846 (49.5%)	85 (40.5%)	0.43 (0.19 to 0.97), *p* = 0.041	0.85 (0.37–1.96), *p* = 0.704
Gross Household Income				
<6000 CHF/month	292 (17.1%)	36 (17.1%)	reference	
6000–9000 CHF/month	351 (20.5%)	40 (19.0%)	0.84 (0.46 to 1.56), *p* = 0.586	0.79 (0.41–1.51), *p* = 0.471
>9000 CHF/month	602 (35.2%)	60 (28.6%)	0.54 (0.31 to 0.92), *p* = 0.025	0.49 (0.26–0.94), *p* = 0.032
Does not know/refuses to say	195 (11.4%)	29 (13.8%)	1.17 (0.61 to 2.27), *p* = 0.632	1.37 (0.67–2.8), *p* = 0.384
Lifestyle factors				
BMI				
<18.5 kg/m^2^	48 (2.8%)	2 (1.0%)	0.62 (0.11–3.55), *p* = 0.588	0.61 (0.1–3.69), *p* = 0.589
18.5–24 kg/m^2^	999 (58.5%)	64 (30.5%)	reference	reference
25–29 kg/m^2^	493 (28.8%)	86 (41.0%)	2.61 (1.66–4.12), *p* < 0.001	4.62 (2.82–7.56), *p* < 0.001
≥30 kg/m^2^	169 (9.9%)	58 (27.6%)	4.89 (2.94–8.12), *p* < 0.001	6.55 (3.77–11.38), *p* < 0.001
Self-Reported Physical Activity				
Not meeting WHO recommendations	675 (39.5%)	89 (42.4%)	reference	
Meeting WHO recommendations	1018 (59.6%)	119 (56.7%)	0.83 (0.57 to 1.22), *p* = 0.343	0.76 (0.5–1.15), *p* = 0.196
Smoking Status				
Never or former	776 (45.4%)	80 (38.1%)	reference	
Current	930 (54.4%)	129 (61.4%)	1.33 (0.91 to 1.96), *p* = 0.144	1.47 (0.95–2.26), *p* = 0.083
Alcohol Consumption				
No or low alcohol consumption	452 (26.4%)	73 (34.8%)	reference	
Higher consumption	1257 (73.6%)	137 (65.2%)	0.69 (0.47 to 1.02), *p* = 0.06	0.61 (0.38–0.97), *p* = 0.037
Meat Consumption				
no	35 (2.0%)	3 (1.4%)	reference	
yes	1674 (98.0%)	207 (98.6%)	2.37 (0.69 to 8.09), *p* = 0.17	3.85 (0.46–32.49), *p* = 0.216
Eating Habits				
≤4 meals outside home	892 (52.2%)	118 (56.2%)	reference	
>4 meals outside home	817 (47.8%)	92 (43.8%)	0.91 (0.63 to 1.32), *p* = 0.63	1.13 (0.67–1.89), *p* = 0.653

Abbreviation: CHF: Swiss franc, BMI: body mass index, WHO: World Health Organisation.

**Table 3:** Predictors of protein intake below dietary reference values (DRV).

	Protein Intake above DRV	Protein Intake below DRV		
	N = 1597	N = 322		
Sociodemographic factors				
			OR univariate (95% CI), *p*-value	OR multivariate (95% CI, *p*-value
Age				
18–29 years	335 (21.0%)	39 (12.1%)	reference	
30–44 years	409 (25.6%)	61 (18.9%)	1.09 (0.63–1.89), *p* = 0.76	0.88 (0.48–1.59), *p* = 0.668
45–59 years	519 (32.5%)	77 (23.9%)	1.03 (0.62–1.71), *p* = 0.9	0.83 (0.47–1.47), *p* = 0.519
60–75 years	334 (20.9%)	145 (45.0%)	3.91 (2.42–6.31), *p* < 0.001	2.94 (1.57–5.52), *p* = 0.001
Sex				
Male	773 (48.4%)	127 (39.4%)	reference	
Female	824 (51.6%)	195 (60.6%)	1.32 (0.97–1.8), *p* = 0.08	1.73 (1.15–2.6), *p* = 0.008
Nationality				
Non-Swiss	220 (13.8%)	24 (7.5%)	reference	
Swiss	1377 (86.2%)	298 (92.5%)	1.63 (0.98–2.71), *p* = 0.06	1.23 (0.72–2.12), *p* = 0.45
Language Region				
German speaking	1036 (64.9%)	213 (66.1%)	reference	
French Speaking	396 (24.8%)	77 (23.9%)	0.95 (0.68–1.32), *p* = 0.741	1.01 (0.68–1.51), *p* = 0.948
Italian Speaking	165 (10.3%)	32 (9.9%)	0.99 (0.59–1.67), *p* = 0.982	1.04 (0.51–2.13), *p* = 0.917
Household Type				
Without Children	990 (62.0%)	224 (69.6%)	reference	
With Children	605 (37.9%)	97 (30.1%)	0.57 (0.41–0.78), *p* < 0.001	0.94 (0.62–1.43), *p* = 0.776
Marital Status				
Not married	735 (46.0%)	133 (41.3%)	reference	
Married	860 (53.9%)	188 (58.4%)	1.03 (0.76–1.4), *p* = 0.83	1.07 (0.69–1.67), *p* = 0.753
Socioeconomic factors				
Education, Highest Degree				
Primary	67 (4.2%)	14 (4.3%)	reference	
Secondary	737 (46.1%)	167 (51.9%)	1.19 (0.59–2.42), *p* = 0.629	1.18 (0.55–2.52), *p* = 0.667
Tertiary	791 (49.5%)	140 (43.5%)	1.11 (0.54–2.28), *p* = 0.769	1.27 (0.6–2.71), *p* = 0.53
Gross Household Income				
<6000 CHF/month	270 (16.9%)	58 (18.0%)	reference	
6000–9000 CHF/month	311 (19.5%)	80 (24.8%)	1.29 (0.81–2.06), *p* = 0.286	1.35 (0.8–2.28), *p* = 0.267
>9000 CHF/month	565 (35.4%)	97 (30.1%)	0.76 (0.49–1.17), *p* = 0.207	1.02 (0.59–1.75), *p* = 0.939
Does not know/refuses to say	194 (12.1%)	30 (9.3%)	0.59 (0.34–1.05), *p* = 0.071	0.8 (0.43–1.48), *p* = 0.477
Lifestyle factors				
BMI				
<18.5 kg/m^2^	46 (2.9%)	4 (1.2%)	0.21 (0.07–0.61), *p* = 0.004	0.11 (0.02–0.53), *p* = 0.006
18.5–24 kg/m^2^	923 (57.8%)	140 (43.5%)	reference	reference
25–29 kg/m^2^	450 (28.2%)	129 (40.1%)	1.76 (1.25–2.46), *p* = 0.001	1.88 (1.23–2.89), *p* = 0.004
≥30 kg/m^2^	178 (11.1%)	49 (15.2%)	1.57 (1.02–2.42), *p* = 0.041	1.38 (0.83–2.3), *p* = 0.22
Self-Reported Physical Activity				
Not meeting WHO recommendations	636 (39.8%)	128 (39.8%)	reference	
Meeting WHO recommendations	945 (59.2%)	192 (59.6%)	1.13 (0.83–1.54), *p* = 0.43	1.01 (0.72–1.42), *p* = 0.937
Smoking Status				
Never or former	713 (44.6%)	143 (44.4%)	reference	
Current	881 (55.2%)	178 (55.3%)	0.86 (0.63–1.16), *p* = 0.323	0.91 (0.64–1.29), *p* = 0.583
Alcohol Consumption				
No or low alcohol consumption	420 (26.3%)	105 (32.6%)	reference	
Higher consumption	1177 (73.7%)	217 (67.4%)	0.74 (0.54–1.02), *p* = 0.07	0.63 (0.42–0.93), *p* = 0.019
Meat Consumption				
no	23 (1.4%)	15 (4.7%)	reference	
yes	1574 (98.6%)	307 (95.3%)	0.27 (0.11–0.64), *p* < 0.001	0.23 (0.1–0.53), *p* = 0.001
Eating habits				
≤4 meals outside home	797 (49.9%)	213 (66.1%)	reference	
>4 meals outside home	800 (50.1%)	109 (33.9%)	0.53 (0.39–0.73), *p* < 0.001	0.95 (0.63–1.43), *p* = 0.802

Abbreviation: CHF: Swiss franc, BMI: body mass index, WHO: World Health Organisation.

## Data Availability

Restrictions apply to the availability of these data. Data was obtained from the Center for Primary Care and Public Health (Unisanté), University of Lausanne, Switzerland (Unisanté), Swiss Federal Food Safety and Veterinary Office (FSVO)] and are available at [https://menuch.unisante.ch/index.php/catalog] (accessed on 1 May 2023) with the permission of the study initiators.

## Supplementary Materials

The following supporting information can be downloaded at: https://www.mdpi.com/article/10.3390/nu15092200/s1, Supplementary Table S1. Energy below resting metabolic rate stratified by gender; Supplementary Table S2. Protein below daily reference value stratified by age groups; Supplemetary Table S3. High protein intake (>2 g/d); Supplementary Figure S1. Protein source in participants with protein intake above and below DRV.

## References

[1] Society G.N. Referenzwerte fur die Nahrstoffzufuhr/Reference Values for Nutrient Intake 2017. https://www.dge.de/wissenschaft/referenzwerte/?L=0.

[2] World Health Organization (2020). Healthy Diet. https://www.who.int/news-room/fact-sheets/detail/healthy-diet.

[3] Phillips J.A. (2021). Dietary Guidelines for Americans, 2020–2025. Workplace Health Saf..

[4] (2022). European Food Safety Authority. https://www.efsa.europa.eu/en/topics/topic/dietary-reference-values.

[5] Dr. Bucher Della Torre Sophie DJCC. Project “Nutritional Reference Values (NRVs) for Switzerland”. Federal Food Safety and Veterinary Office FSVO Contract No 0714001567. June 2021. https://www.blv.admin.ch/dam/blv/de/dokumente/lebensmittel-und-ernaehrung/ernaehrung/nutri-score/bericht-naehrwertreferenzwerte-schweiz.pdf.download.pdf/Full%20report_NRV_HEdS_final.pdf.

[6] Schuetz P., Seres D., Lobo D.N., Gomes F., Kaegi-Braun N., Stanga Z. (2021). Management of disease-related malnutrition for patients being treated in hospital. Lancet.

[7] Cederholm T., Barazzoni R., Austin P., Ballmer P., Biolo G., Bischoff S.C., Compher C., Correia I., Higashiguchi T., Holst M. (2017). ESPEN guidelines on definitions and terminology of clinical nutrition. Clin. Nutr..

[8] Hanna K.L., Collins P.F. (2015). Relationship between living alone and food and nutrient intake. Nutr. Rev..

[9] Dumartheray E.W., Krieg M.A., Cornuz J., Whittamore D.R., Lanham-New S.A., Burckhardt P. (2006). Energy and nutrient intake of Swiss women aged 75–87 years. J. Hum. Nutr. Diet..

[10] Marques-Vidal P., Rousi E., Paccaud F., Gaspoz J.M., Theler J.M., Bochud M., Stringhini S., Guessous I. (2015). Dietary Intake according to Gender and Education: A Twenty-Year Trend in a Swiss Adult Population. Nutrients.

[11] Si Hassen W., Castetbon K., Cardon P., Enaux C., Nicolaou M., Lien N., Terragni L., Holdsworth M., Stronks K., Hercberg S. (2016). Socioeconomic Indicators Are Independently Associated with Nutrient Intake in French Adults: A DEDIPAC Study. Nutrients.

[12] van Rossum C.T., van de Mheen H., Witteman J.C., Grobbee E., Mackenbach J.P. (2000). Education and nutrient intake in Dutch elderly people. The Rotterdam Study. Eur. J. Clin. Nutr..

[13] Chatelan A., Marques-Vidal P., Bucher S., Siegenthaler S., Metzger N., Zuberbühler C.A., Camenzind-Frey E., Reggli A., Bochud M., Beer-Borst S. (2017). Lessons Learnt about Conducting a Multilingual Nutrition Survey in Switzerland: Results from menuCH Pilot Survey. Int. J. Vitam. Nutr. Res..

[14] Switzerland-National Nutrition Survey menuCH 2014–2015. Center for Primary Care and Public Health (Unisanté), University of Lausanne, Switzerland (Unisanté), Swiss Federal Food Safety and Veterinary Office (FSVO). 1 September 2022. https://https://menuch.unisante.ch/index.php/catalog/4.

[15] Mifflin M.D., St Jeor S.T., Hill L.A., Scott B.J., Daugherty S.A., Koh Y.O. (1990). A new predictive equation for resting energy expenditure in healthy individuals. Am. J. Clin. Nutr..

[16] Deutsche Gesellschaft für Ernährung, Österreichische Gesellschaft für Ernährung (2019). Schweizerische Gesellschaft für Ernährung, Hrsg. Referenzwerte für die Nährstoffzufuhr.

[17] Babor T.F., Higgins-Biddle J.C. (2001). Brief Intervention for Hazardous and Harmful Drinking: A Manual for Use in Primary Care.

[18] Bull F.C., Al-Ansari S.S., Biddle S., Borodulin K., Buman M.P., Cardon G., Carty C., Chaput J.P., Chastin S., Chou R. (2020). World Health Organization 2020 guidelines on physical activity and sedentary behaviour. Br. J. Sport. Med..

[19] Hagströmer M., Oja P., Sjöström M. (2006). The International Physical Activity Questionnaire (IPAQ): A study of concurrent and construct validity. Public Health Nutr..

[20] Pasquier J., Chatelan A., Bochud M. (2017). Weighting Strategy.

[21] Bauer J., Biolo G., Cederholm T., Cesari M., Cruz-Jentoft A.J., Morley J.E., Phillips S., Sieber C., Stehle P., Teta D. (2013). Evidence-based recommendations for optimal dietary protein intake in older people: A position paper from the PROT-AGE Study Group. J. Am. Med. Dir. Assoc..

[22] Tieland M., Borgonjen-Van den Berg K.J., Van Loon L.J., de Groot L.C. (2015). Dietary Protein Intake in Dutch Elderly People: A Focus on Protein Sources. Nutrients.

[23] Tieland M., Borgonjen-Van den Berg K.J., van Loon L.J., de Groot L.C. (2012). Dietary protein intake in community-dwelling, frail, and institutionalized elderly people: Scope for improvement. Eur. J. Nutr..

[24] Pirlich M., Schütz T., Kemps M., Luhman N., Minko N., Lübke H.J., Rossnagel K., Willich S.N., Lochs H. (2005). Social risk factors for hospital malnutrition. Nutrition.

[25] Hone M., Nugent A.P., Walton J., McNulty B.A., Egan B. (2020). Habitual protein intake, protein distribution patterns and dietary sources in Irish adults with stratification by sex and age. J. Hum. Nutr. Diet..

[26] Houston D.K., Nicklas B.J., Ding J., Harris T.B., Tylavsky F.A., Newman A.B., Lee J.S., Sahyoun N.R., Visser M., Kritchevsky S.B. (2008). Dietary protein intake is associated with lean mass change in older, community-dwelling adults: The Health, Aging, and Body Composition (Health ABC) Study. Am. J. Clin. Nutr..

[27] Tschanz L., Kaelin I., Wróbel A., Rohrmann S., Sych J. (2022). Characterisation of meat consumption across socio-demographic, lifestyle and anthropometric groups in Switzerland: Results from the National Nutrition Survey menuCH. Public Health Nutr..

[28] Steinbach L., Rohrmann S., Kaelin I., Krieger J.P., Pestoni G., Herter-Aeberli I., Faeh D., Sych J. (2021). No-meat eaters are less likely to be overweight or obese, but take dietary supplements more often: Results from the Swiss National Nutrition survey menuCH. Public Health Nutr..

[29] van Vliet S., Burd N.A., van Loon L.J. (2015). The Skeletal Muscle Anabolic Response to Plant- versus Animal-Based Protein Consumption. J. Nutr..

[30] Gorissen S.H.M., Witard O.C. (2018). Characterising the muscle anabolic potential of dairy, meat and plant-based protein sources in older adults. Proc. Nutr. Soc..

[31] Traversy G., Chaput J.P. (2015). Alcohol Consumption and Obesity: An Update. Curr. Obes. Rep..

[32] Cancello R., Soranna D., Brunani A., Scacchi M., Tagliaferri A., Mai S., Marzullo P., Zambon A., Invitti C. (2018). Analysis of Predictive Equations for Estimating Resting Energy Expenditure in a Large Cohort of Morbidly Obese Patients. Front. Endocrinol..

[33] Woo J., Leung S.S., Ho S.C., Sham A., Lam T.H., Janus E.D. (1999). Influence of educational level and marital status on dietary intake, obesity and other cardiovascular risk factors in a Hong Kong Chinese population. Eur. J. Clin. Nutr..

[34] Haste F.M., Brooke O.G., Anderson H.R., Bland J.M., Peacock J.L. (1990). Social determinants of nutrient intake in smokers and non-smokers during pregnancy. J. Epidemiol. Community Health.

[35] Pearson J.M., Schlettwein-Gsell D., van Staveren W., de Groot L. (1998). Living alone does not adversely affect nutrient intake and nutritional status of 70- to 75-year-old men and women in small towns across Europe. Int. J. Food Sci. Nutr..

[36] Lee K.W., Shin D. (2021). Comparison of Dietary Behaviors and the Prevalence of Metabolic Syndrome in Single- and Multi-Person Households among Korean Adults. Healthcare.

[37] Alkerwi A., Baydarlioglu B., Sauvageot N., Stranges S., Lemmens P., Shivappa N., Hébert J.R. (2017). Smoking status is inversely associated with overall diet quality: Findings from the ORISCAV-LUX study. Clin. Nutr..

[38] Dallongeville J., Marécaux N., Fruchart J.C., Amouyel P. (1998). Cigarette smoking is associated with unhealthy patterns of nutrient intake: A meta-analysis. J. Nutr..

[39] Dyer A.R., Elliott P., Stamler J., Chan Q., Ueshima H., Zhou B.F. (2003). Dietary intake in male and female smokers, ex-smokers, and never smokers: The INTERMAP study. J. Hum. Hypertens..

[40] Hughes J.R., Hatsukami D.K. (1997). Effects of three doses of transdermal nicotine on post-cessation eating, hunger and weight. J. Subst. Abus..

[41] Pestoni G., Krieger J.P., Sych J.M., Faeh D., Rohrmann S. (2019). Cultural Differences in Diet and Determinants of Diet Quality in Switzerland: Results from the National Nutrition Survey menuCH. Nutrients.

[42] Inanir D., Kaelin I., Pestoni G., Faeh D., Mueller N., Rohrmann S., Sych J. (2021). Daily and meal-based assessment of dairy and corresponding protein intake in Switzerland: Results from the National Nutrition Survey menuCH. Eur. J. Nutr..

[43] Friel S., Kelleher C.C., Nolan G., Harrington J. (2003). Social diversity of Irish adults nutritional intake. Eur. J. Clin. Nutr..

[44] Crispim S.P., de Vries J.H., Geelen A., Souverein O.W., Hulshof P.J., Lafay L., Rousseau A.S., Lillegaard I.T., Andersen L.F., Huybrechts I. (2011). Two non-consecutive 24 h recalls using EPIC-Soft software are sufficiently valid for comparing protein and potassium intake between five European centres--results from the European Food Consumption Validation (EFCOVAL) study. Br. J. Nutr..

[45] Chatelan A., Beer-Borst S., Randriamiharisoa A., Pasquier J., Blanco J.M., Siegenthaler S., Paccaud F., Slimani N., Nicolas G., Camenzind-Frey E. (2017). Major Differences in Diet across Three Linguistic Regions of Switzerland: Results from the First National Nutrition Survey menuCH. Nutrients.

[46] SwissVeg. https://www.swissveg.ch/2021_10_Anzahl_Veganer_Vegetarier?language=de.

[47] Langsetmo L., Harrison S., Jonnalagadda S., Pereira S.L., Shikany J.M., Farsijani S., Lane N.E., Cauley J.A., Stone K., Cawthon P.M. (2020). Low Protein Intake Irrespective of Source is Associated with Higher Mortality among Older Community-dwelling Men. J. Nutr. Health Aging.

[48] Coelho-Junior H.J., Marzetti E., Picca A., Cesari M., Uchida M.C., Calvani R. (2020). Protein Intake and Frailty: A Matter of Quantity, Quality, and Timing. Nutrients.

