# Supplementary material for: Association of Sociodemographic, Socioeconomic and Lifestyle Characteristics with Low Protein and Energy Intake in the Healthy Swiss Population
Source: Nutrients. 2023 May 5;15(9):2200. doi: 10.3390/nu15092200 (PMC10181371; doi:10.3390/nu15092200)
Supplement: Supplementary file 1 [file nutrients-15-02200-s001.zip › nutrients-2367835-supplementary.pdf]

nutrients

MDPI

Supplementary Table S1. Energy below resting metabolic rate stratified by gender

	female	male
Sociodemographic factors		
	OR multivariate (95% CI, p-value	OR multivariate (95% CI, p-value
Age		
18-29 years	reference	
30-44 years	0.44 (0.16-1.21), p=0.112	1.42 (0.47-4.26), p=0.53
45-59 years	0.45 (0.17-1.17), p=0.101	1.59 (0.54-4.64), p=0.398
60-75 years	0.29 (0.08-1.06), p=0.062	1.04 (0.3-3.66), p=0.951
Nationality		
Non- Swiss	reference	
Swiss	0.3 (0.13-0.7), p=0.005	1.03 (0.47-2.22), p=0.947
Language Region		
German speaking	reference	
French Speaking	1.48 (0.76-2.86), p=0.245	0.98 (0.45-2.15), p=0.968
Italian Speaking	1.16 (0.41-3.27), p=0.778	1.71 (0.83-3.51), p=0.146
Household Type		
Without Children	reference	
With Children	2.1 (1.15-3.85), p=0.016	0.91 (0.48-1.74), p=0.779
Marital Status		
Not married	reference	
Married	0.97 (0.49-1.93), p=0.93	1.09 (0.52-2.28), p=0.814
Socioeconomic factors		
Education, Highest Degree		
Primary	reference	
Secondary	0.88 (0.26-2.93), p=0.829	0.55 (0.19-1.61), p=0.276
Tertiary	0.55 (0.15-2.04), p=0.372	0.77 (0.28-2.12), p=0.612
Gross Household Income		
<6000CHF/month	reference	
6000-9000CHF/month	0.78 (0.26-2.35), p=0.658	0.69 (0.3-1.58), p=0.383
>9000CHF/month	0.95 (0.35-2.59), p=0.919	0.27 (0.12-0.62), p=0.002
Does not know/refuses to say	1.59 (0.51-4.94), p=0.424	1.21 (0.42-3.46), p=0.72

Lifestyle factors		
BMI		
<18.5 kg/m2	0.73 (0.13-4.03), p=0.722	empty
18.5-24 kg/m2	reference	reference
25-29 kg/m2	4.74 (2.47-9.13), p=0	5.61 (2.45-12.82), p=0
≥ 30 kg/m2	5.95 (2.8-12.63), p=0	8.45 (3.45-20.71), p=0
Self-Reported Physical Activity		
Not meeting WHO recommendations	reference	
Meeting WHO recommendations	0.8 (0.43-1.52), p=0.501	0.76 (0.44-1.31), p=0.32
Smoking Status		
Never or	reference	
Current	1.12 (0.6-2.08), p=0.725	2.01 (1.05-3.84), p=0.034
Alcohol Consumption		
No or low alcohol consumption	reference	
Higher consumption	0.55 (0.3-1.02), p=0.059	0.64 (0.31-1.32), p=0.228
Meat Consumption		
No	reference	
Yes	2.11 (0.24-18.32), p=0.5	omitted (predicts failure perfectly)
Eating Habits		
≤ 4 meals outside home	reference	
> 4 meals outside home	0.94 (0.48-1.83), p=0.847	1.16 (0.55-2.46), p=0.694

Abrev.: CHF: Swiss franc, BMI: body mass index, WHO: World Health Organisation

Supplementary Table S2. Protein below daily reference value stratified by age groups

	18-29 years	30-44 years	45-59 years	60-75 years
Sociodemographic factors				
	OR multivariate (95% CI, p-value	OR multivariate (95% CI, p-value	OR multivariate (95% CI, p-value	OR multivariate (95% CI, p-value
Sex				
Male	reference			
Female	3.2 (0.71-14.52), p=0.131	3.73 (1.63-8.53), p=0.002	1.42 (0.65-3.06), p=0.378	1.04 (0.55-1.98), p=0.902
Nationality				
Non- Swiss	reference			
Swiss	0.63 (0.19-2.13), p=0.459	1.09 (0.42-2.82), p=0.862	1.83 (0.56-5.92), p=0.315	1.1 (0.37-3.29), p=0.858
Language Region				
German speaking	reference			
French Speaking	3.16 (1.06-9.39), p=0.038	0.31 (0.1-0.96), p=0.043	1.07 (0.5-2.25), p=0.868	1.22 (0.68-2.19), p=0.497
Italian Speaking	0.28 (0.03-2.78), p=0.275	1.16 (0.29-4.62), p=0.829	2.24 (0.69-7.35), p=0.181	0.73 (0.29-1.85), p=0.506
Household Type				
Without Children	reference			
With Children	0.79 (0.19-3.3), p=0.748	0.58 (0.25-1.37), p=0.215	1.63 (0.83-3.17), p=0.154	0.34 (0.11-1.03), p=0.057
Marital Status				
Not married	reference			
Married	0.4 (0.09-1.82), p=0.237	1.86 (0.71-4.89), p=0.205	0.9 (0.41-1.97), p=0.794	1.16 (0.6-2.27), p=0.659
Socioeconomic factors				

Education, Highest Degree				
Primary	reference			
Secondary	1.93 (0.19-19.8), p=0.579	*	0.98 (0.19-5.02), p=0.977	0.75 (0.25-2.2), p=0.599
Tertiary	1.39 (0.14-13.44), p=0.778	*	0.59 (0.11-3.1), p=0.532	1.06 (0.35-3.18), p=0.914
Gross Household Income				
<6000CHF/month	reference			
6000-9000CHF/month	2.83 (0.58-13.71), p=0.195	2.01 (0.37-10.79), p=0.415	0.51 (0.19-1.42), p=0.198	1.44 (0.67-3.07), p=0.349
>9000CHF/month	2.77 (0.55-13.96), p=0.216	1.81 (0.33-9.83), p=0.494	0.57 (0.23-1.45), p=0.24	0.55 (0.24-1.27), p=0.164
Does not know/refuses to say	0.85 (0.14-5.24), p=0.861	0.99 (0.15-6.73), p=0.991	0.16 (0.04-0.69), p=0.014	1.25 (0.51-3.07), p=0.62
Lifestyle factors				
BMI				
<18.5 kg/m2	*	*	1.44 (0.12-17.14), p=0.773	0.04 (0-3.56), p=0.158
18.5-24 kg/m2	reference	reference		
25-29 kg/m2	0.37 (0.08-1.71), p=0.204	4.21 (1.8-9.83), p=0.001	2.58 (1.18-5.64), p=0.018	1.21 (0.61-2.4), p=0.581
≥ 30 kg/m2	2.82 (0.76-10.48), p=0.121	0.27 (0.06-1.26), p=0.096	1.15 (0.34-3.86), p=0.816	1.64 (0.72-3.7), p=0.237
Self-Reported Physical Activity				
Not meeting WHO recommendations	reference			

Meeting WHO recommendations	2.52 (0.85-7.43), p=0.094	1.82 (0.81-4.08), p=0.147	0.55 (0.28-1.09), p=0.086	0.9 (0.52-1.54), p=0.69
Smoking Status				
Never or	reference			
Current	0.84 (0.28-2.49), p=0.746	1.69 (0.77-3.74), p=0.193	0.65 (0.32-1.3), p=0.225	0.87 (0.5-1.49), p=0.6
Alcohol Consumption				
No or low alcohol consumption	reference			
Higher consumption	1.1 (0.41-2.96), p=0.852	0.63 (0.29-1.39), p=0.25	0.44 (0.19-1.01), p=0.052	0.56 (0.29-1.09), p=0.09
Meat Consumption				
No	reference			
Yes	0.1 (0.02-0.47), p=0.004	0.71 (0.15-3.37), p=0.662	0.26 (0.03-1.97), p=0.192	0.04 (0-0.42), p=0.008
Eating habits				
≤ 4 meals outside home	reference			
> 4 meals outside home	0.41 (0.16-1.06), p=0.066	0.9 (0.37-2.24), p=0.827	0.92 (0.44-1.94), p=0.836	1.64 (0.69-3.87), p=0.263

Abrev.: WHO:World Health Organisation

* omitted because predicts failure perfectly

Supplemetary Table S3. High protein intake (>2g/d).

Sociodemographic factors	Protein intake above DRV N=1811	Protein intake below DRV N=108	OR univariate (95% CI), p-value	OR multivariate (95% CI, p-value
Age				
18-29 years	333 (18.4%)	41 (38.0%)	reference	
30-44 years	446 (24.6%)	24 (22.2%)	0.65 (0.31-1.40), p=0.27	0.52 (0.27-1.00), p=0.05
45-59 years	569 (31.4%)	27 (25.0%)	0.61 (0.33-1.12), p=0.11	0.33 (0.15-0.76), p=0.009
60-75 years	463 (25.6%)	16 (14.8%)	0.37 (0.16-0.83), p=0.02	0.34 (0.11-1.10), p=0.07
Sex				
Male	829 (45.8%)	71 (65.7%)	reference	
Female	982 (54.2%)	37 (34.3%)	0.63 (0.34-1.16), p=0.14	0.51 (o.26-0.10), p=0.05
Nationality				
Non- Swiss	229 (12.6%)	15 (13.9%)	reference	
Swiss	1582 (87.4%)	93 (86.1%)	0.74 (0.35-1.54), p=0.42	0.76 (0.33-1.71), p=0.49
Language Region				
German speaking	1191 (65.8%)	58 (53.7%)	reference	
French Speaking	444 (24.5%)	29 (26.9%)	1.12 (0.64-1.97), p=0.69	1.40 (0.76-2.61), p=0.28
Italian Speaking	176 (9.7%)	21 (19.4%)	1.78 (0.96-3.31), p=0.07	2.02 (0.90-4.52), p=0.09
Household Type				
Without Children	1142 (63.1%)	72 (66.7%)	reference	
With Children	666 (36.8%)	36 (33.3%)	1.19 (0.66-2.15), p=0.56	1.32(0.67-2.63), p=0.42
Marital Status				
Not married	812 (44.8%)	56 (51.9%)	reference	

Married	996 (55.0%)	52 (48.1%)	1.20 (0.70-2.05), p=0.50	1.54 (0.74-3.24), p=0.25
Socioeconomic factors				
Education, Highest Degree				
Primary	70 (3.9%)	11 (10.2%)	reference	
Secondary	848 (46.8%)	56 (51.9%)	0.33 (0.13-0.81), p=0.02	0.38 (0.16-0.90), p=0.03
Tertiary	890 (49.1%)	41 (38.0%)	0.30 (0.11-0.79), p=0.02	0.34 (0.13-0.89), p=0.03
Gross Household Income				
<6000CHF/month	306 (16.9%)	22 (20.4%)	reference	
6000-9000CHF/month	376 (20.8%)	15 (13.9%)	0.74 (0.24-2.29), p=0.60	0.75 (0.25-2.25), p=0.61
>9000CHF/month	631 (34.8%)	31 (28.7%)	0.73 (0.36-1.47), p=0.38	0.66 (0.29-1.45), p=0.30
Does not know/refuses to say	202 (11.2%)	22 (20.4%)	1.10 (0.52-2.34), p=0.81	0.71 (0.33-1.52), p=0.38
	296 (16.3%)			
Lifestyle factors				
BMI				
<18.5 kg/m2	42 (2.3%)	8 (7.4%)	2.11 (0.80-5.52), p=0.13	3.64 (1.14-11.64), p=0.03
18.5-24 kg/m2	999 (55.2%)	64 (59.3%)	reference	reference
25-29 kg/m2	555 (30.6%)	24 (22.2%)	0.55 (0.30-1.01), p=0.05	0.43 (0.20-0.90), p=0.03
≥ 30 kg/m2	215 (11.9%)	12 (11.1%)	0.68 (0.30-1.53), p=0.35	0.55 (0.23-1.33), p=0.19
Self-Reported Physical Activity				
Not meeting WHO recommendations	721 (39.8%)	43 (39.8%)	reference	
Meeting WHO recommendations	1073 (59.2%)	64 (59.3%)	0.82 (0.46-1.44), p=0.49	0.74 (0.39-1.41), p=0.36
Smoking Status				
Never or former	809 (44.7%)	47 (43.5%)	reference	
Current	998 (55.1%)	61 (56.5%)	1.17 (0.65-2.10), p=0.60	0.90 (0.46-1.79), p=0.76

Alcohol Consumption				
No or low alcohol consumption	493 (27.2%)	32 (29.6%)	reference	
Higher consumption	1318 (72.8%)	76 (70.4%)	0.94 (0.54-1.63), p=0.82	1.29 (0.69-2.42), p=0.42
Meat Consumption				
no	37 (2.0%)	1 (0.9%)	reference	
yes	1774 (98.0%)	107 (99.1%)	4.77 (0.63-36.13), p=0.13	omitted
Eating habits				
≤ 4 meals outside home	962 (53.1%)	48 (44.4%)	reference	
> 4 meals outside home	849 (46.9%)	60 (55.6%)	1.30 (0.76-2.23), p=0.34	1.08 (0.56-2.05), p=0.83

Abrev.: WHO:World Health Organisation, DRV: daily reference value

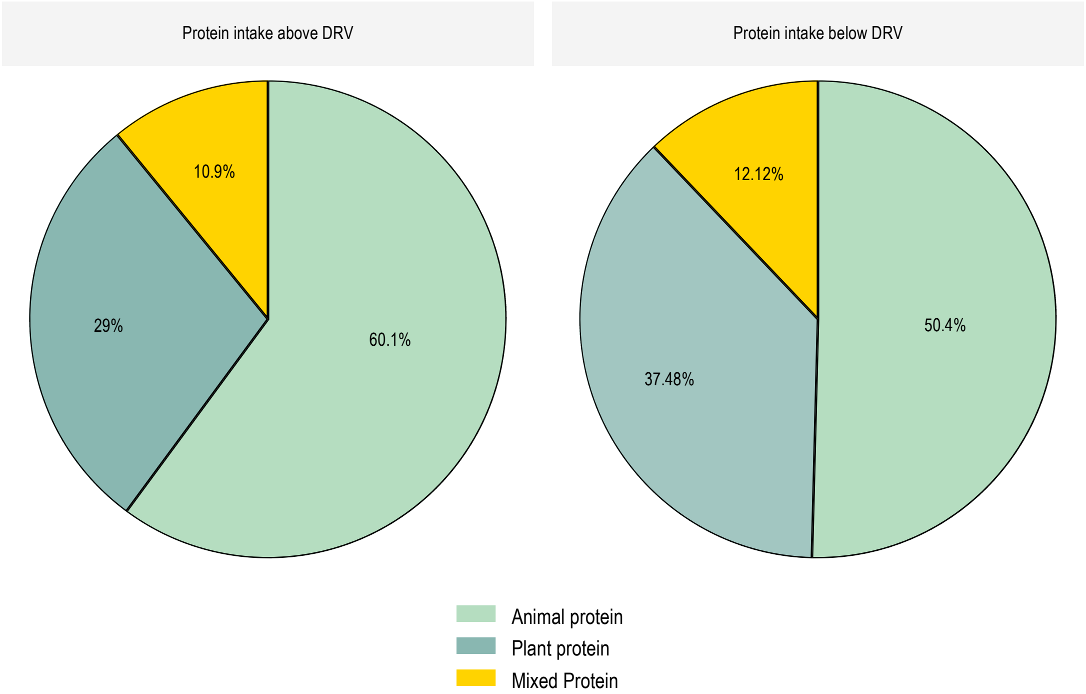

Supplementary Figure S1. Protein source in participants with protein intake above and below DRV.
Protein sources were determined by two study authors and categorized into three groups (animal-based, plant-based, mixed). Food products with unclear protein origins or animal- and plant-based proteins were classified as mixed.
